# Accumulation of metallic trace elements in *Reynoutria japonica*: a risk assessment for plant biomass valorization

**DOI:** 10.1007/s11356-022-20485-7

**Published:** 2022-05-06

**Authors:** Sylvain Lerch, Catherine Sirguey, Alice Michelot-Antalik, Stefan Jurjanz

**Affiliations:** 1 Université de Lorraine, INRAE, URAFPA, 54000 Nancy, France; 2 Ruminant Research Group, Agroscope, 1725 Posieux, Switzerland; 3 Université de Lorraine, INRAE, 54000 Nancy, LSE France; 4 LAE, Université de Lorraine, INRAE, 54000 Nancy, France

**Keywords:** Asian *R. japonica* (syn. *Fallopia* spp.), Biological invasion, Cadmium, Environmental safety, Transfer soil–plant, Bioaccumulation, Roughage, Organic amendment

## Abstract

Sustainable solutions aiming at limiting *Reynoutria japonica* invasion consist of frequent removal of its aerial biomass. The aims of this study were to measure the accumulation of metallic trace elements (MTE) in *R. japonica,* and to assess the eco-toxicological risk related to the valorization of the produced biomass. *R. japonica* fragmented rhizomes were regenerated in pots for 41 days on a control soil (CTL) or a moderately MTE-contaminated soil (POL, 3.6 mg Cd kg^−1^ DM). Growth traits were recorded, as well as MTE bioconcentration (BCF) and translocation factors (TF) from soil to plant organs. Whatever the MTE and plant organs, BCF remained below one (mean Cd-BCF for stem and leaf: 0.07 and 0.29 for CTL and POL, respectively), conversely to TF (until 2.2 for Cd and Ni in POL soil). When grown on the POL soil, *R. japonica* stem and leaf Cd content was close to the EU maximum regulatory limit for organic amendments or animal feed. Model simulations suggested that liver and kidney Cd concentrations would exceed the regulatory limit in food when adult cattle or sheep constantly ingest *R. japonica* grown on the POL soil over 200 to 800 days. The results of the present study will be useful to help managers in selecting efficient and safe solutions for the control of *R. japonica* invasion.

**Supplementary Information:**

The online version contains supplementary material available at 10.1007/s11356-022-20485-7.

## Introduction

*Reynoutria japonica* Houtt., 1777 (Japanese knotweed; syn. *Fallopia japonica var. japonica*, Polygonaceae), was introduced in 1840 in the Netherlands for ornamental, melliferous, and fodder uses (Bailey and Conolly 2000; Djeddour and Shaw 2010). Population dynamics of *R. japonica* has been exponential since 1950 in Europe and recovers a wide range of habitats among which localities along roads and water courses (Mandák et al. 2004). *R. japonica* is among the most invasive plant species in north-western Europe (Pyšek et al. 2001), with a fragmentation and a dispersion of rhizomes mainly by floods or human activity. As such, *R. japonica* is part of the list of invasive species established by the European Union to coordinate efforts of containing their propagation (European Commission 2014, 2016, 2017). Different plant characteristics promote their biological invasion. *R. japonica* has a long growing cycle (from March to November) with a fast growth up to 3 m of height. It is a geophyte plant with highly resistant rhizomes up to 3 m deep and great capacities of dispersion and regeneration: rhizome fragments of 1 cm length and 0.7 g weight being able to regenerate (Bailey et al. 2009). The plant is highly competitive for nitrogen (Aguilera et al. 2010) and its rhizomes could exudate allelopathic substances which inhibit germination and growth of competitor plants (e.g. Pinzone et al. 2018; Vrchotová and Šerá 2008). Moreover, in urban or industrial conditions, *R. japonica* is able to spread on soil with various pH ranges, and nutrient and pollutant content (Sołtysiak and Brej 2014). Such an invasion has become a real issue for managers of public or private spaces and is today an often-cited example to illustrate the different damages by invasive plants: ecologic disorders, damages on infrastructure and roads, and increased risks of jam and flood of water courses and of accidents due to reduced visibility (Gover et al. 2005; Mclean 2010; Payne and Hoxley 2012). The financial costs due to *R. japonica* invasions can be very high. Indeed, Reinhardt et al. (2003) estimated to 32 million euro for 2200 ha the cost of *R. japonica* control in Germany including direct damages, control methods, and maintenance. In England, Williams et al. (2010) estimated to 180 million euro the global cost of negative effects of *R. japonica*.

The two main ways to limit and reduce expansion of *R. japonica* are mechanical (plant cutting, barriers, roots digging out) and chemical treatments (glyphosate spraying), their use in combination being the more efficient (Bailey et al. 2009; Mclean 2010). Nonetheless, the use of chemical treatments become progressively restricted because of the environmental health risks concomitant of their use and managers are looking nowadays for more sustainable control solutions. Among them, an efficient strategy aiming at limiting and reducing *R. japonica* invasion consists in frequent removal and exportation of its aerial biomass (i.e. stems and leaves) over several years. Such medium- to long-term strategy allows progressively reducing *R. japonica* growth (Michelot-Antalik et al. 2016). This biomass removal can be performed, thanks to mechanical cutting and further composting or methanization of the biomass exported (Boyer and Barthod 2019), or through livestock grazing (Lelay et al. 2019; Lerch et al. 2016). However, the development of the latter two strategies may be compromised in case of metallic trace elements (MTE) contamination of the aerial biomass of *R. japonica* growing on soil-polluted fields. On the one hand, the land spread of *R. japonica* compost or digestate may embody a relevant input of MTE into agricultural soils used for crop production, and further compromise the chemical safety of vegetable food chain. On the other hand, in case of livestock grazing, ingestion over several years of *R. japonica* leaves and stems containing high levels of MTE may endanger not only animal health, but also safety of food of animal origin (i.e. liver and kidney, Lane et al. 2015). Besides, many studies have shown that *R. japonica* is able to grow on highly anthropized soils (e.g. industrial wasteland, settlement area or roadsides) sometimes highly contaminated in MTE (Berchová-Bímová et al. 2014; Širka et al. 2016; Rahmonov et al. 2014, 2019; Sołtysiak et al. 2011). In these studies, the soil metal concentrations were in the range: 0.05–6.75 mg Cd kg^−1^, 5.8–203 mg Pb kg^−1^, and 10.5–2623 mg Zn kg^−1^. Most of these articles reported that *R. japonica* seems able to accumulate MTE with the highest concentrations of metals in rhizome, whereas the uncontrolled conditions of such field studies did not allow to address to which extent the above-ground organs are supplied by MTE from the rhizome or from air borne contamination (Böhmová and Šoltés 2017). Suggestion of such a translocation of MTE from rhizome to above-ground organs was recently provided by two greenhouse-controlled conditions studies, but observations were limited to Cd, Cr, and Zn and for measurements on either a single (Barberis et al. 2020) or triplicate (Michalet et al. 2017) per treatment pooled samples of stem and leaf together. Therefore, further investigations in controlled conditions quantifying the transfer of MTE from soil to *R. japonica* below- and above-ground organs are needed. Additionally, to the best of the authors’ knowledge, up-to-date MTE risk assessment due to biomass valorization of *R. japonica* growing on polluted soils has never been performed. The objectives of this study were (i) to measure the accumulation of MTE by different organs of *R. japonica* when growing in controlled conditions on a realistically and moderately contaminated soil and (ii) to evaluate the eco-toxicological risk related to the valorization of the produced biomass through modelling approaches, with a focus on Cd as target MTE and methanization, composting and grazing as biomass valorization.

## Materials and Methods

### Soil material and analyses

A pot experiment was conducted with the top horizon (0–20 cm) of an agricultural gleyic Luvisol (IUSS Working Group 2015) located at Chenevières (Grand Est, France). The studied treatments correspond to the natural topsoil (CTL) and the same topsoil with aged artificial pollution (POL). In a previous experiment, a batch of the 2-mm sieved top horizon has been artificially spiked with Cd, Cu, Ni, Pb, and Zn and used for the cultivation of the hyperaccumulator plant *N. caerulescens* over 3 years (2013–2016; Sterckeman et al. 2017, 2019). Then, the polluted soil was air-dried and stored at room temperature before the culture of *R. japonica*. As a control, a new sampling of the top horizon was realized in 2017 at the same place. The soil was air-dried for 2 days, homogenized, and sieved at 2 mm before culture. Soil physico-chemical properties and MTE analyses were performed by the Laboratory of Soil Analyses (Arras, France) according to French standards (AFNOR 2009) and results are reported in Table 1. Granulometric fractions have been determined according to the Robinson pipette method following the NF X31 107 standard. Pseudo-total elements were obtained from the *aqua regia* soil digestion, based on the NF ISO 11464 standard. A test portion of 0.5 g at most was digested with a mixture of 6 mL HCl and 2 mL HNO_3_, then progressively heated to 105 °C for 3 h, using a heating block system (DigiPREP, SCP Science, Quebec, QC, Canada). The final extract was filtered at 0.45 μm and adjusted to 50 ml with water before analysis by ICP-OES (iCAP 6000 series, Thermo Scientific, Cambridge, UK). Total CaCO_3_, organic C, and total N contents were measured by dry combustion of crushed soil (at 250 μm) and quantification of the emitted volume of CO_2_ and N_2_ (NF ISO 10694). The determination of total nitrogen followed the ISO 13878 standard, that of total and organic carbon the ISO 14235 standard, and the determination of total limestone the ISO 10693. To measure water pH, 5.0 ml of soil was added to 25 ml of ultrapure water according to NF ISO 10390. The cation exchange capacity (CEC) and the extraction of the exchangeable cations were implemented according to the NF ISO 23470 standard. Exchangeable cations were then analysed by ICP-OES. Quality control assessment was done by inserting certified control soils as samples. To verify the validity of the ICP-OES results, a blank and a control sample, as well as a certified solution (EU-H3, SCP Science, Courtaboeuf, France), were inserted regularly into the batches to be analysed.

**Table 1:** Physico-chemical properties and metallic trace elements concentrations of the control (CTL) and polluted (POL) soils

Parameters	Units	CTL	POL
*Granulometric fractions*			
Clay (0–2 µm)	%	10.1	10.3
Fine silt (2–20 µm)	%	19.9	19.1
Coarse silt (20–50 µm)	%	13.9	13.9
Fine sand (50–200 µm)	%	15.7	15.2
Coarse sand (200–2000 µm)	%	40.4	41.5
*Nutrient content*			
Total N	%	0.100	0.105
Total P	%	61.2	60.0
Total S	%	< 28.2	41.1
Organic C	%	1.05	0.974
C/N		10.5	9.28
*Acido-basic status*			
pH water		6.74	6.45
CaCO_3_	%	< 0.1	0.12
Effective CEC^a^	cmol^+^ kg^−1^	7.01	6.12
Ca	cmol^+^ kg^−1^	7.69	8.39
K	cmol^+^ kg^−1^	0.165	0.231
Mg	cmol^+^ kg^−1^	0.346	0.354
Na	cmol^+^ kg^−1^	0.306	0.541
*Total metal concentrations (Aqua Regia extraction)*			
Cd	mg kg^−1^	0.37	3.63
Cu	mg kg^−1^	9.5	28.4
Ni	mg kg^−1^	15.2	120
Pb	mg kg^−1^	15.8	60.2
Zn	mg kg^−1^	76.6	649

^a^Cobaltihexammine cationic exchange capacity and exchangeable cations

### Plant material and cultivation

Rhizome fragments originating from individual *R. japonica* shrubs have been sampled up-to 30 cm depth the 30 March 2017 in Vandœuvre-lès-Nancy (Grand Est, France, GPS 48°39′22″ N, 6°09′37″ E) during *R. japonica* budburst. Rhizomes have been stored at 4 °C during 48 h before any treatment. After cleaning the rhizomes from adhering soil with tap water, dead wood was removed and fragments of 8 to 10 cm length were cut. Because of the strong variation of rhizome’s diameter (from 0.8 to 4.2 cm) and weight (from 7.7 to 290 g), fragments have been sorted to form 15 blocks of two, each block showing similar diameter and weight (i.e. 15 replicates divided into 15 blocks within each block one replicate per treatment). Because rooting volume can affect the plant metal extraction (Baklanov et al. 2015), the soil volume was adapted to the rhizome size. Thus, small rhizome couples (i.e. blocks 1 to 13) were planted in 1 L pots filled with 1.2 ± 0.16 kg (mean ± standard deviation) of dry soil. Rhizomes more than 150 g (i.e. blocks 14 and 15) were planted in 3 L pots filled with 3.5 ± 0.3 kg dry soil. This block design allowed replication of each soil treatment per block and for each pot an average ratio of 25 g dry soil per g of rhizome, the rhizome fragments being covered with 2 to 3 cm of soil. After plantation, all 30 pots were placed within a climatic chamber with the following controlled growing conditions: 23 °C/18 °C day/night temperature, 16 h/8 h day/night cycle, 350 µmol photons m^2^ s^−1^ light intensity, and 60% air humidity. Pots were watered with tap water every 2 days by capillarity.

### Growth measurements and sampling

*R. japonica* growth parameters were followed during 41 days by weekly records of the vegetative height and of total leaf number (Pérez-Harguindeguy et al. 2013). At harvest (day 41), vegetative height, total leaf number, and stem diameter have been measured for each plant. For each pot, plant organs (leaves, stem, and rhizomes) were separated and weighed before and after drying at 70 °C for 48 h (dry matter determination, DM). Rhizomes were thoroughly cleaned of all adhering soil with tap water before measurement.

### Plant analyses

Dry biomass of each *R. japonica* organ was ground using an agate mortar before the digestion of a 0.5 g test portion with 8 mL 65% HNO_3_ for 12 h and 4 mL 30% H_2_O_2_ for 3.5 h at 95 °C, using the DigiPREP® system (SCP Science, Baie-d’Urfé, QC, Canada). After filtration of the extract at 0.45 μm, the samples were adjusted to 25 mL with deionized water (18 MΩ). Major nutrients (Ca, K, Mg, Na, S, P) and transition metals (Cd, Cu, Fe, Mn, Ni, and Zn) in the plant extracts were determined by induced coupled plasma emission spectrometry (ICP-AES, iCAP 6000 Series, Thermo Scientific, Cambridge, UK). To check the validity of the results, a blank and a control sample, as well as a certified solution (EU-H3, SCP Science, Courtaboeuf, France), were inserted regularly into the batches to be analysed.

### Calculations and statistical analyses

The bioconcentration factors (BCF) were calculated, for a given MTE, as the ratio of its concentration (mg kg^−1^ DM) in *R. japonica* organs (rhizome, stem, or leaf) to its pseudo-total concentration (mg kg^−1^ DM) in the soil. Translocation factors (TF) were calculated as the ratio of the MTE concentration in stem to rhizome (translocation from rhizome to stem), and in leaf to stem (translocation from stem to leaf).

All data were analysed by ANOVA using the MIXED procedure of SAS (version 9.4, SAS Institute Inc., Cary, NC, USA, 2003) with a model including the type of soil (CTL and POL) as a fixed effect, and the block as a random effect. Additionally, for vegetative height and total leaf number, which were measured in kinetic along the experimental period, the day and the interaction soil × day were also included as fixed effects, and the day as a repeated statement. For MTE concentrations in *R. japonica* organs, the organ (rhizome, stem, and leaf) and the interaction soil × organ were included as fixed effects, and the organ as a repeated statement. For BCF and TF, the MTE (Cd, Cu, Ni, and Zn) and the interaction soil × MTE were included as fixed effects, and the MTE as a repeated statement. An autoregressive first order covariance structure was used, at the exception of vegetative height and total leaf number for which a spatial power covariance structure was used (in order to take into account the unequal time intervals between measurements). Logarithmic transformation of experimental data was performed whenever needed (i.e. for *R. japonica* organs Cd, Ni, Al, Fe, and Mn concentrations, the three BCF and the stem/leaf TF) to comply with the assumptions of normality and homoscedasticity of residuals. In this case, least squares means and standard errors of the mean (SEM) were calculated from untransformed values, whereas declared *P*-values reflect statistical analysis of transformed data. Differences were declared significant at *P* ≤ 0.05. Trends towards significance were considered at 0.05 < *P* ≤ 0.10. Values reported are least square means and SEM.

### Estimates of Cd transfer from grazed *R. japonica* to ruminant offal

In case of *R. japonica* grazing, among the four MTE for which transfer factors were produced thank to the present study (i.e. Cd, Cu, Ni, and Zn), a priority was given to the assessment of the risks of Cd transfer to the feed and animal food chain. Estimations of Cd transfer from *R. japonica* stems and leafs orally ingested into kidney and liver of adult cattle (constant body weight (BW) of 600 kg) or sheep (constant BW of 60 kg) were computed using four different modelling approaches (cattle: Franz et al. 2008; Römkens et al. 2008; sheep: Beresford et al. 1999; Prankel et al. 2004). The use of two models by species allows to reduce uncertainty due to their complementary approaches. For cattle, Franz et al. (2008) proposed the use of linear accumulation equations based on biotransfer rates (BTR) from feed ingestion to kidney and liver (latter called “Franz model”), assuming an irreversible Cd accumulation without elimination from the target organ. Additionally, Römkens et al. (2008) introduced a steady state model for kidney (latter called “Römkens model”), assuming that Cd not only accumulate but is excreted as well. An exponential equation then describes the relationship between Cd intake and Cd levels in the organs. For sheep, Prankel et al. (2004) proposed the use of empirical relationships based on a meta-analysis of 21 animal experimentations and 90 treatments (latter called “Prankel model”). In such instance, kidney or liver Cd concentrations are derived, thanks to linear regressions depending on diet Cd concentration and time of exposure. Lastly, Beresford et al. (1999) fitted a multi-compartment model describing dynamically the fate of Cd from oral intake to diverse sheep organs and tissues, including kidney and liver (latter called “Beresford model”), from which specific biotransfer factors (BTF) after 100 or 1000 days of exposure were derived and further used. Full details of the model equations are provided in the Supplementary Material S1.

For Franz, Römkens and Beresford model estimations, Cd daily ingestion was needed and computed from the average concentration of Cd in *R. japonica* stem and leaf multiplied by the DM intake (DMI, kg day^−1^), which was estimated thanks to the French INRA feeding system for ruminant (INRA 2018).

For adult cattle:

1 $$\mathrm{DMI }({\mathrm{kg\,d}}^{-1})=[3.2 + 0.015 \times \mathrm{ BW }(\mathrm{kg})]/\mathit{R. japonica}\ \mathrm{fill \,unit}$$

For adult sheep:

2 $$\mathrm{DMI }({\mathrm{kg \,d}}^{-1})=0.081\times {\mathrm{BW}}^{0.75} (\mathrm{kg})/\mathit{R. japonica}\ \mathrm{fill \,unit}$$

where an average fill unit of 1 kg^−1^ DM for *R. japonica* leaf and stem was assumed as reported by Lerch et al. (2016).

Further estimations of Cd transfer from *R. japonica* stems and leaves into offal of cattle or sheep were computed according the four models. The four scenarios tested were a constant feeding of *R. japonica* over 5.5 years at the following Cd concentrations:

i) CTL: 0.025 mg kg^−1^ DM (mean of *R. japonica* stem and leaf growing on CTL soil),
ii) POL: 1.05 mg kg^−1^ DM (mean of *R. japonica* stem and leaf growing on POL soil),
iii) *R. japonica* growing on the median urban, industrial, traffic, mining, and military areas (SUITMA) French topsoils (1.30 mg kg^−1^ DM, Joimel et al. 2016) — lower bound estimation (SUITMA lb): 0.09 mg kg^−1^ DM (mean soil to leaf and stem BCF of 0.07 recorded for CTL),
iv) *R. japonica* growing on the median SUITMA French topsoils — upper bound estimation (SUITMA ub): 0.38 mg kg^−1^ DM (mean soil to leaf and stem BCF of 0.29 recorded for POL).

## Results

### Plant growth parameters

**Fig. 1:**
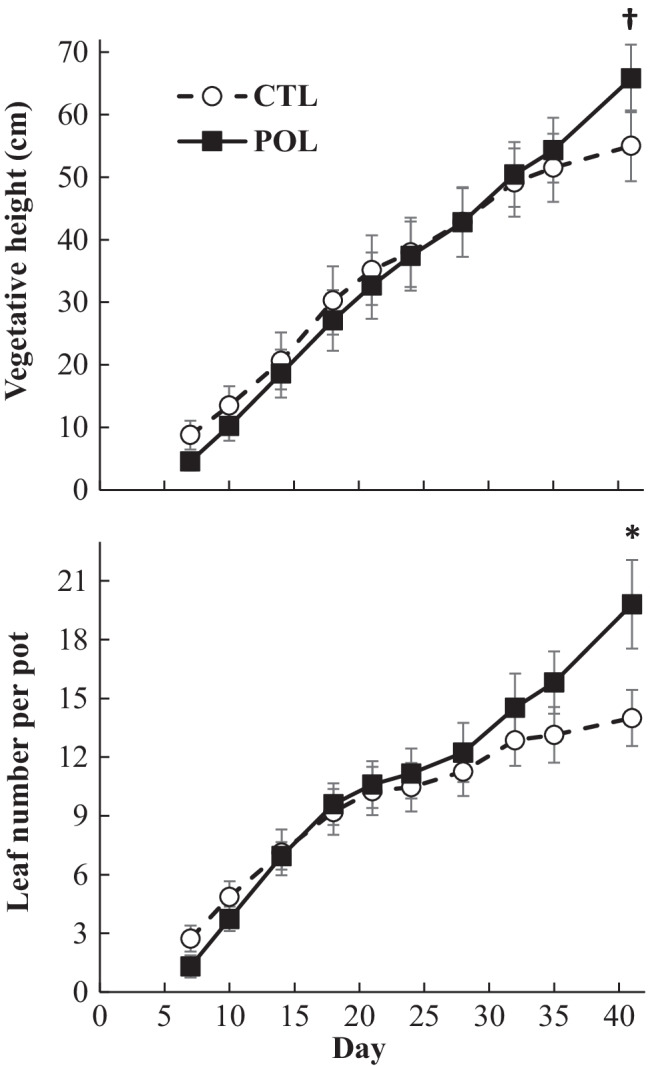
Time pattern of the vegetative height and number of leaves per pot of *R. japonica* cultivated on a control (CTL) or metallic trace elements polluted (POL) soil. Results are given as least square means with the standard error of the mean as error bars. Significant levels for soil effect on the considered day: † (*P* ≤ 0.10), * (P ≤ 0.05)

Kinetic of vegetative height and total leaf number over the course of the experiment are reported in Fig. 1 and details of growth parameters at harvest in Table 2. Whatever the type of soil, both vegetative height and leaf number increased steadily and linearly at average daily rates of 1.5 ± 0.5 cm and 0.42 ± 0.19 leaf over the 41 days of experiment. Over the course of the experiment, the type of soil had no effect on those growth parameters, except at the end (day 41) where leaf number was higher (*P* < 0.05) and vegetative height tended to be higher (*P* < 0.10) for POL than for CTL soil (Fig. 1). Nonetheless, none fresh and dry organs biomasses were affected by the type of soil (*P* > 0.10), even if stem and leaf dry matter contents were lower (*P* < 0.05) for POL than for CTL soil (Table 2).

**Table 2:** Growth parameters at harvest of *R. japonica* cultivated 41 days on a control (CTL) or metallic trace elements polluted (POL) soil^a^

	CTL		POL		*P*-value
*Rhizome*					
Dry mass (g)	20.0	*4.7*	18.7	*4.7*	0.55
Dry matter content (%)	29.6	*1.2*	29.2	*1.3*	0.80
*Stem*					
Height (cm)	55.0	*5.7*	65.8	*5.4*	0.09
Diameter (cm)	0.9	*0.1*	0.9	*0.1*	0.98
Dry mass (g)	3.7	*0.8*	4.2	*0.8*	0.70
Dry matter content (%)	29.1	*1.3*	23.7	*0.9*	< 0.01
*Leaf*					
Number	16.9	*2.0*	28.9	*4.2*	0.02
Dry mass (g)	7.1	*1.0*	7.5	*0.9*	0.72
Dry matter content (%)	28.4	*2.7*	22.7	*1.0*	0.02

^a^Results are given as least square means with the standard error of the mean in italic and *P*-value for soil effect

### Plant MTE concentrations

**Fig. 2:**
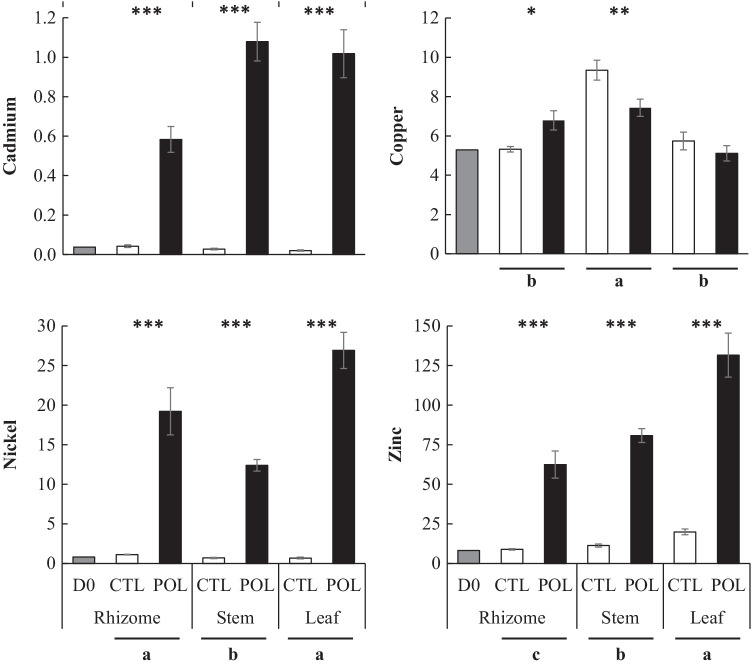
Cadmium, copper, nickel, and zinc concentrations (mg kg^−1^ dry matter) in organs of *R. japonica* at the end of the experiment after growing up for 41 days on control (CTL, black filled bars) or metallic trace elements polluted (POL, open bars) soil. Concentrations for rhizomes at the beginning of the experiment are also reported (day 0: D0, grey filled bars). Each bar represents treatment least square means with standard error of the mean as error bar. Significant levels for soil effect on the considered organ are represented by *, **, and *** symbols (*P* ≤ 0.05, 0.01, and 0.001, respectively). Significant levels for organ effect (means for organ concentration pooled across soils) at *P* ≤ 0.05 are represented with different letters

Concentrations of Cd, Cu, Ni, and Zn in *R. japonica* organs are reported in Fig. 2, their BCF from soil to *R. japonica* organs and TF from rhizome to stem and from stem to leaf are presented in Table 3, whereas details of metal concentrations are available in Supplementary Table S1. Among the five MTE spiked in the POL soil, only Pb was not detected in either *R. japonica* rhizome, stem, or leaf (concentration lower than the limit of quantification of 1.4 mg kg^−1^ DM). Copper concentrations were only slightly affected by either type of soil or organ, as they did not differ from more than 1.8 order of magnitude between leaf of POL soil and stem of CTL soil (Fig. 2). Conversely, Zn, Ni, and Cd concentrations in all *R. japonica* organs were higher (*P* < 0.01) for POL than for CTL soil: for Zn, sevenfold in rhizome, stem, and leaf; for Ni, 18-fold in rhizome and stem and 38-fold in leaf; and for Cd, 15, 36 and 51-fold in rhizome, stem and leaf, respectively. Overall, when *R. japonica* was cultivated on POL soil, leaf was the organ that concentrated the more the MTE except for Cu that concentrated the more in stem. However, different behaviours were observed depending of the MTE. Thus, the highest concentrations of Cd were found in both stem and leaf with values about 1 mg kg^−1^ which was 1.7-fold higher than rhizome concentration. For Ni, the concentrations varied from 12.4 to 26.9 mg kg^−1^ with the highest value in leaf and the lowest in stem whereas Zn concentrations increased gradually from rhizome (62.4 mg kg^−1^) to leaf (131 mg kg^−1^).

**Table 3:** Bioconcentration and translocation factors for cadmium, copper, nickel, and zinc in *R. japonica* at the end of the experiment after growing up for 41 days on control (CTL) or metallic trace elements polluted (POL) soil^a^

Metallic trace elements	Cadmium		Copper		Nickel		Zinc			*P*-value		
Soil	CTL	POL	CTL	POL	CTL	POL	CTL	POL	SEM	Metal	Soil	Metal × Soil
*Bioconcentration factor*^*b*^												
Rhizome/soil	0.01d	0.16b	0.19ab	0.24a	0.01d	0.16b	0.01d	0.09c	0.011	< 0.001	< 0.001	< 0.001
Stem/soil	0.08de	0.30b	0.98a	0.26b	0.05e	0.10cd	0.15cd	0.12c	0.013	< 0.001	< 0.01	< 0.001
Leaf/soil	0.06d	0.28b	0.60a	0.18c	0.05d	0.23bc	0.26bc	0.22bc	0.015	< 0.001	< 0.001	< 0.001
*Translocation factor*^*c*^												
Stem/rhizome	0.81cd	2.15a	1.77ab	1.20bcd	0.62d	0.96cd	1.31bc	1.68ab	0.149	< 0.001	0.07	< 0.001
Leaf/stem	0.95bc	0.95bc	0.62c	0.69c	1.15b	2.18a	1.79a	1.82a	0.061	< 0.001	< 0.001	< 0.001

^a^Results are given as least square means. Different letters within a row indicate significant differences at *P* ≤ 0.05

^b^Bioconcentration factors are defined as metallic trace element concentration in *R. japonica* organ (rhizome, stem, or leaf) divided by total concentration in soil (concentrations in mg kg^−1^ dry matter)

^c^Stem/rhizome and leaf/stem translocation factors are defined as metallic trace element concentration in stem or leaf divided by concentration in rhizome or stem, respectively (concentrations in mg kg^−1^ dry matter)

When considering the BCF, two groups of MTE were observed: Cu and Zn on the one hand, and Cd and Ni on the other hand (Table 3). The particularity of the Cu–Zn group is that BCF_stem_ and BCF_leaf_ were higher in CTL than in POL soil, whereas the reverse was observed for BCF_rhizome_. Moreover, whatever the considered organ, BCF were higher (*P* < 0.05) for Cu (from 0.19 to 0.98) than for Zn (from 0.01 to 0.26). In Cd-Ni group, whatever the considered organ, BCF were higher (*P* < 0.05) in POL than in CTL soil with values ranging from 0.1 (Ni-BCF_rhizome_) to 0.3 (Cd-BCF_leaf_) without any significant difference between MTE except for BCF_stem_. The difference between these two groups is less pronounced when looking at the TF (Table 3). However, one can mention the absence of soil effect in the Cu–Zn group (*P* > 0.10), whereas a significant effect (*P* < 0.05) of soil was observed for leaf/stem Ni-TF and for stem/rhizome Cd-TF with higher values in POL than in CTL soil. Moreover, for both Cu and Zn, stem/rhizome TF showed a tendency to decrease in POL soil. Globally, whatever the organ, MTE, and type of soil, translocation factors were always higher than 0.6. The highest values were found for stem/rhizome Cu-TF and leaf/stem Zn-TF (1.8) in CTL soil and for leaf/stem Ni-TF (2.2) in POL soil.

### Estimates of Cd transfer from grazed *R. japonica* to ruminant offal

**Fig. 3:**
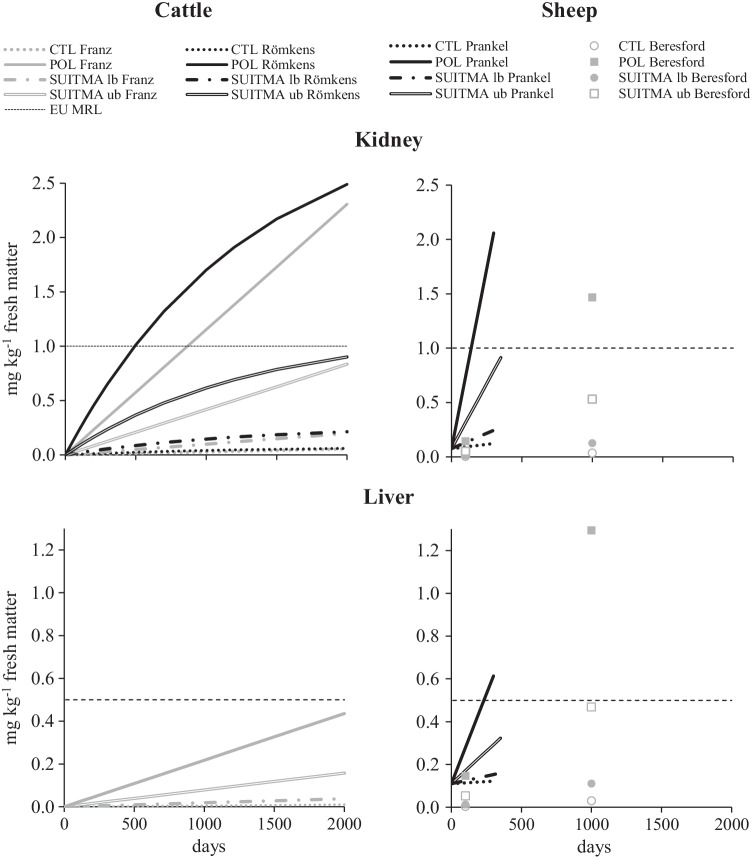
Predictions of kidney (upper panels) and liver (lower panels) Cd levels from transfer models for adult cattle (left panels) or sheep (right panels) fed constantly with *R. japonica* growing on the control (CTL) or polluted (POL) experimental soils, or on a soil with a Cd level of 1.30 mg kg^−1^ DM (median of SUITMA French soils distribution, Joimel et al., 2016) with lower or upper bounds (SUITMA lb and ub, respectively) soil to *R. japonica* bioconcentration factor scenarios. Cattle linear model: Franz et al. 2008 or steady-state model: Römkens et al. 2008. Sheep empirical model: Prankel et al. 2004 or dynamic model: Beresford et al. 1999

Kinetic simulations of kidney and liver Cd concentrations according to the several scenarios and models are reported in Fig. 3. For cattle, kidney simulations were in close accordance for both the linear Franz model and the steady-state Römkens model and suggest that only constant 500 to 800 days ingestion of *R. japonica* growing on the POL soil would lead to kidney Cd concentration above 1.0 mg kg^−1^ fresh matter (FM). Conversely, for liver, a Cd concentration of 0.5 mg kg^−1^ FM would not be achieved even after 2000 days for the POL soil. Besides, after 2000 days, kidney Cd concentration would attain 0.83 and 0.90 mg kg^−1^ FM for Franz and Römkens models, respectively in case of the median SUITMA upper bound scenario. For sheep, kidney and liver Cd concentrations would be above 1.0 and 0.5 mg kg^−1^ after 140 and 240 days, respectively, according to the Prankel model, as well as at 1,000 days according to the fixed-time BTF derived from the Beresford model. Liver Cd concentration would also reach a level of 0.47 mg kg^−1^ at 1000 days for *R. japonica* growing on a French median SUITMA topsoil and using an upper bound BCF scenario.

## Discussion

### Soil properties and MTE concentrations

As expected, both soils present very similar physico-chemical properties. Only pH, total organic carbon, and cationic exchange capacity were slightly lowered in POL than in CTL soil due to the repeated cultivation without fertilization. The level of pollution in soils can be assessed by using some statistical indicators (vibriss) used as threshold values for the detection of trace element anomalies (Duigou et al. 2011). The dataset, coming from Saby et al. (2018), gives the vibriss values corresponding to the background level in the region where the topsoil has been collected. The respective values for Cd, Cu, Ni, Pb, and Zn are 0.92–70.6–123-74–215 mg kg^−1^. Based on the MTE analyses (Table 1), CTL is not polluted, whereas POL can be considered as a moderately contaminated soil on which *R. japonica* could establish and grow. Indeed, only Cd and Zn concentrations are slightly higher than the contamination limit criteria commonly accepted as three times the vibriss value (Jensen et al. 2006).

### Growth and MTE accumulation in plant organs

In case of the POL treatment, *R. japonica* was cultivated on a polymetallic contaminated soil comparable to urban topsoils moderately contaminated (Joimel et al. 2016; Pouyat et al. 2010). Our results showed that *R. japonica* has a slightly better growth in POL than in CTL soil. The growth difference between soils was detectable from day 25 but significant only at the end of the 41-day period of growth. This phenomenon could indicate that the photosynthetic allocation is not inhibited on POL soil and, an acclimatization of *R. japonica* to the pollution conditions occurred as it was observed in *Lagerstroemia speciosa* (Sulistijorini et al. 2008). When cultivated in controlled conditions (Cd range: 0.1–10 mg kg^−1^, Cr range: 23.4–332 mg kg^−1^, and Zn range: 42.9–924 mg Zn kg^−1^), other authors found evidence that the presence of MTE in soil has a low impact on *Reynoutria* spp. overall growth traits during rhizome regeneration, and has a rather stimulating effect on plant growth depending on pollution level, which may enhance their competitiveness in relation to other plant species (Barberis et al. 2020; Michalet et al. 2017). Indeed, according to these studies, Cr and to a lesser extent also Zn could stimulate the plant growth. It is hypothesized that such a positive effect could be mediated by the production of secondary metabolites by *R. japonica* and reflect plant root interactions with rhizosperic microbes. Those molecules could be directly or indirectly involved in plant tolerance and adaptation to metallic stress (Barberis et al. 2020; Michalet et al. 2017).

Numerous on-field studies about the metal transfer in Japanese *R. japonica* are available in literature but the very different on-field conditions limit their comparison. Several authors (Berchová-Bímová et al. 2014; Rahmonov et al. 2014, 2019; Širka et al. 2016) studied the metal transfer into *R. japonica* in urban contaminated sites with soil MTE concentrations in the range 0.14–6.75 mg Cd kg^−1^, 2.7–89.7 mg Cu kg^−1^, 12.3–178 mg Pb kg^−1^, and 41.5–581 mg Zn kg^−1^. The POL soil used in the present study falls within this range of concentrations. Similarly, the concentrations of MTE measured in the present study in the different plant organs of *R. japonica* were quite low and in the range of those measured in these former studies. In *R. japonica* leaves, the Cd concentration (1.02 mg kg^−1^) was slightly above those measured in plants in France (0–1 mg Cd kg^−1^), whereas both Ni (26.9 mg kg^−1^) and Zn (131.5 mg kg^−1^) concentrations were respectively 5 and 3 times higher (Tremel-Schaub and Feix, 2005). If Cd, Ni, and Zn have been bioaccumulated in all organs, BCF were all below one showing a low ability of the species to accumulate MTE in such soil conditions. Only one study mentioned that *Polygonum cuspidatum* growing in metalliferous habitats (mines and smelters surrounding areas) was able to accumulate up to 12.2 mg Cd kg^−1^ and 1614 mg Zn kg^−1^ in leaves and 50.4 mg Cd kg^−1^ and 5040 mg Zn kg^−1^ in roots with BCF still below one.

To the best of the authors’ knowledge, only Michalet et al. (2017) and Barberis et al. (2020) previously investigated MTE transfer into *R. japonica* growing for three months in controlled conditions on freshly spiked soils with Cd (0.1–10 mg kg^−1^), Cr (9–440 mg kg^−1^), Pb (6–295 mg kg^−1^), and Zn (18–880 mg kg^−1^). If Barberis et al. (2020) confirmed the very low BCFs for Cd (< 0.3), that for Zn reached 1.4. Besides, Michalet et al. (2017) obtained Zn-BCF_shoot_ between 0.5 and 0.8 and Cd-BCF_shoot_ from 1 to 3. The present results on soils with similar Cd and Zn concentrations than in Michalet et al. (2017) did not confirm such high BCFs. Such discrepancies may be due to the use of freshly spiked soils in Barberis et al. (2020) and Michalet et al. (2017), experiments contrarily to the present study in which soil contamination was aged for several years, leading to a decrease of MTE bioavailability by complexation reaction, especially with organic matter (Denys et al. 2007). The same phenomena may also explain the differences observed between BCF_rhizome_ in the present study (Table 3: all BCF < 0.3) and those reported by Barberis et al. (2020) for Zn (BCF between 1 and 2), Cr (BCF between 1 and 8), and Cd (BCF up to 15). Besides, the study of a different *Reynoutria* spp. (i.e., *R* × *Bohemica* in Barberis et al. 2020 and Michalet et al. 2017, vs. *R. Japonica* in the present study) and accordingly phenotype diversity, probably also modulate the rate of MTE transfer from soil to plant.

Thus, the present study suggests that *R. japonica* only slightly accumulate MTE in their above-ground tissues when growing on a soil moderately contaminated. Nonetheless, as the MTE concentrations achieved in the POL Japanese *R. japonica* are above normal values, it is mandatory to assess the eco-toxicological risk related to the valorization of the produced biomass.

### Consequences for valorization of *R. japonica* biomass

During composting or methanization, an increase of around 1.2 to 1.4 fold of the MTE concentrations on DM basis from *R. japonica* organs cutting to compost or digestate would occur due to the losses (i.e. fermentation and volatilization) of part of the organic matter, and further increase in the concentration of inorganic matter in DM (Francou et al. 2008). When such fold increase is applied for the POL experimental treatment, MTE concentrations in *R. japonica* compost or methanization digestate still comply with the French regulations for organic amendments safety (NF U44-051/A2; AFNOR 2018). However, leaf and stem Cd and leaf Ni are close from the 1.5 and 50 mg kg^−1^ DM limits set in the European End of Waste criteria project (Saveyn and Eder 2014). When using the BCF from POL soil to leaf or stem of 0.29 for Cd and 0.23 for Ni, the maximum limits proposed in the End of Waste criteria project would be outreached for soil over 3.7 mg Cd kg^−1^ DM and 155 mg Ni kg^−1^ DM. For Cd, such 3.7 mg kg^−1^ DM level is exceeded in around one third of SUITMA French topsoils (Joimel et al. 2016), which constitute typical sites where Japanese *R. japonica* may expand (Michalet et al. 2017; Rahmonov et al. 2014). Nevertheless, such concentrations were not observed in an European scale survey (Tóth et al. 2016). Conversely, for Ni, this 155 mg kg^−1^ DM level was not reached in the survey of Joimel et al. (2016), but has been observed in topsoils of North-Western Italy and Northern Greece (Tóth et al. 2016).

The assessment of the risk of Cd transfer from grazed *R. japonica* to the feed and animal food chain was evaluated in priority. Indeed, the highest BCF and TF were recorded for this element. Additionally, European authorities set maximum regulatory levels (MRL) in feed and food for Cd (EU No. 1275/2013 amending Directive 2002/32/EC, and EU No. 629/2008 amending Directive 1881/2006, respectively), conversely to Cu, Ni, and Zn for which none specific regulation is fixed. The plant was harvested at 41 days of growth at a height of around 70 cm, a physiological stage that corresponded to usual practices of eco-grazing (Lerch et al. 2016). Nonetheless, observed concentrations could differ when plants of different stages would be grazed by animals. Cadmium concentrations in leaf and stem of *R. japonica* growing on the CTL soil were between 40- and 60-fold below the European feed regulatory limit set at 1.14 mg kg^−1^ DM. Conversely, when growing on POL soil, stem, and leaf Cd contents were close to (1.08 and 1.02 mg kg^−1^ DM, respectively) but still in accordance with this regulatory limit. Using the BCF computed for the POL experimental treatment, such regulatory limit would be outreached in *R. japonica* stem at soil Cd concentrations of 3.8 mg kg^−1^ DM. Cattle model simulations suggested further that constant 500- to 800-day ingestion of *R. japonica* on POL soil would lead to kidney Cd concentration above the EU MRL (1.0 mg kg^−1^ FM), but not the one in liver (0.5 mg kg^−1^ FM, EU No. 629/2008 amending Directive 1881/2006). Moreover, kidney Cd concentration would also be close from the regulatory limit after 2000 days for the median SUITMA upper bound scenario. For sheep, kidney and liver Cd concentrations would be higher than the MRL after 140 and 240 days, respectively, according to the Prankel et al. (2004) empirical relationships, as well as at 1000 days according to the fixed-time BTF derived from the model of Beresford et al. (1999). Liver Cd concentration would also reach a level close from the MRL at 1000 days for *R. japonica* growing on a French median SUITMA topsoil and using an upper bound BCF scenario. For either cattle or sheep, such models estimates did not take into account the fact that in practical situations animal would probably not constantly grazed *R. japonica* on Cd-contaminated area (e.g. above the French SUITMA median). Intermittent periods would obviously be included over the animal lifespan where they would receive feedstuffs less contaminated in Cd, which may buffer the risk of reaching the regulatory limit in offal. Nonetheless, in case soil had a Cd concentration equal or higher than the third quartile of the French SUITMA distribution (34 mg kg^−1^ DM, Joimel et al. 2016), all models lead to the estimation that only 50 to 300 days of *R. japonica* intake would be sufficient to exceed the MRL in cattle or sheep liver and kidney.

## Conclusions

This controlled-condition study quantified the transfer rate of Cd, Cu, Zn, and Ni from control and moderately polluted soils into *R. japonica* rhizome, stem, and leaf, and further provided a risk assessment for the valorization of *R. japonica* biomass through methanization, composting or livestock grazing. When compared to the CTL soil, the growth traits of *R. japonica* growing on the POL soil were not reduced and even slightly improved at the end of the experiment (41 days). Bioconcentration factors from soil to *R. japonica* organs were higher for Cd and Ni in POL than in CTL soil, whereas they were comparable or lower in the case of Cu and Zn. Nonetheless, BCF always remain below 1, conversely to TF of Cd, Cu, and Zn from rhizome to stem and that of Ni and Zn from stem to leaf (until 2.2 in POL soil for Cd from rhizome to stem and for Ni from stem to leaf). When applying such transfer rates of Cd from soil to *R. japonica* biomass as a basis for risk assessment, stem and leaf methanization digestate, compost, or roughage would no more comply with French and EU MRL for MTE in organic amendments or feed when *R. japonica* grow on a soil above 3.7 mg Cd kg^−1^ DM. Moreover, model simulations suggested that kidney or liver Cd concentrations would be higher than the EU MRL for MTE in food of animal origin when adult cattle or sheep ingest *R. japonica* leaf and stem growing on such a 3.7 mg Cd kg^−1^ DM soil constantly over 200 to 800 days (depending on livestock species, offal, and transfer model used). As this soil Cd contamination level is almost never encountered in forest and agricultural soils, in such cases, the *R. japonica* biomass valorization seems safe regarding the risk of Cd transfer to the environment and food chain. Nonetheless, for specific MTE contaminated sites (i.e. urban, industrial, traffic, mining, and military areas: SUITMA), this soil Cd level may be exceeded (e.g. in around one third of the French SUITMA soils). As *R. japonica* may typically grow and expand on such ruderalized sites, the results of the present study will be useful in order to guide and aware managers in finding the best trade-off between the benefit of reducing *R. japonica* invasion and risk of MTE transfer when choosing and implementing specific control solutions.

## Supplementary Information

Below is the link to the electronic supplementary material.

Supplementary file1 (DOCX 30 KB)

## Data Availability

Data can be accessed from the authors by reasonable request.
