# Supplementary material for: Accumulation of metallic trace elements in Reynoutria japonica: a risk assessment for plant biomass valorization
Source: Environ Sci Pollut Res Int. 2022 May 6;29(44):67390–401. doi: 10.1007/s11356-022-20485-7 (PMC9492613; doi:10.1007/s11356-022-20485-7)
Supplement: Supplementary file 1 — Supplementary file1 (DOCX 30 KB) [file 11356_2022_20485_MOESM1_ESM.docx]

**Accumulation of metallic trace elements in *Reynoutria japonica*: a risk assessment for plant biomass valorization**

Sylvain Lerch^1,2,*^, Catherine Sirguey^3^, Alice Michelot-Antalik^4^, Stefan Jurjanz^1^

^1^ Université de Lorraine, INRAE, URAFPA, F-54000 Nancy, France

^2^ Ruminant Research Group, Agroscope, 1725 Posieux, Switzerland

^3^ Université de Lorraine, INRAE, LSE, F-54000 Nancy, France

^4^ Université de Lorraine, INRAE, LAE, F-54000, Nancy, France

*Corresponding author:

Sylvain Lerch, Agroscope, Ruminant Research Group, Route de la Tioleyre 4, CH-1725 Posieux, Switzerland, Tel :+41 58 461 41 29, email : [sylvain.lerch@agroscope.admin.ch](mailto:sylvain.lerch@agroscope.admin.ch)

**Supplementary Material S1**

**Supplementary Table S1**

**Pages S1-S5**

**Supplementary Material S1**

**Details of model equations used for estimation of Cd transfer from grazed *R. japonica* to ruminant offal**

**Franz cattle model**

Franz et al. (2008) proposed the use of linear accumulation equations based on biotransfer rates (BTR) from feed ingestion to kidney and liver, assuming an irreversible Cd accumulation without elimination from the target organ:

*Organ Cd (mg kg^-1^ fresh mass, FM) = BTR (kg^-1^) × Cd daily ingestion (mg d^-1^) × time (t, d) [1]*

With BTR equal to 9.0 × 10^-5^ and 1.7 × 10^-5^ for kidney and liver respectively, and *t* the duration of Cd exposure.

**Römkens cattle model**

Römkens et al. (2008) introduced a steady state model for kidney, assuming that Cd not only accumulate but is excreted as well. An exponential equation then describes the relationship between Cd intake and Cd levels in the organs:

*Kidney Cd (mg kg^-1^ FM) = Kidney Cd_0_ × exp ^- λ × t^ + Kidney Cd_ss_ × (1 – exp ^- λ × t^) [2]*

With, Kidney Cd_0_ (mg kg^-1^ FM) the Cd concentration in kidney at day 0, start level of the simulated period of time (set at 0 in further simulations). Kidney Cd_ss_ (mg kg^-1^ FM) is the steady state concentration, and λ the elimination time constant (day^-1^), which were defined as:

*Kidney Cd_ss_ (mg kg^-1^ FM) = Kidney biotransfer factor (BTF, d kg^-1^) × Cd daily uptake (mg d^-1^) [3]*

*λ (d^-1^) = ln(2) / half life time (d) [4]*

With,

*Kidney BTF (d kg^-1^) = Kidney carry-over rate (%) / [Kidney fresh weight (kg) × λ (d^-1^)] [5]*

*Cd daily uptake (mg d^-1^) = Cd daily ingestion (mg d^-1^) × Cd absorption rate (%) [6]*

Half-life time was fitted to 900 d, carry-over rate to 0.12% and Cd absorption rate to 20% (Römkens et al. 2008), whereas kidney fresh weight was set to 1.26 kg according to Lin et al. (2020; 0.21% BW in a 600 kg BW adult cattle).

**Prankel sheep model**

Prankel et al. (2004) proposed the use of the following empirical relationships based on a meta-analysis of 21 animal experimentations and 90 treatments:

*Kidney Cd (mg kg^-1^ DM) = 0.36 + 0.030 × [R. japonica Cd (mg kg^-1^ DM) × time (t, d)] [7]*

*Liver Cd (mg kg^-1^ DM) = 0.44 + 0.0064 × [R. japonica Cd (mg kg^-1^ DM) × time (t, d)] [8]*

Organ Cd concentrations were further converted on FM basis using DM content of 21 and 25% for kidney and liver, respectively (Phillips and Tudoreanu 2011). Such relationships were not used over more than 350 days of Cd exposure, as only 5 on 90 experimental treatments included in the meta-analysis were of longer duration.

**Beresford sheep model**

Beresford et al. (1999) fitted a dynamic compartmental model of Cd fate into sheep, from which specific BTF for kidney and liver after 100 or 1000 days of exposure were derived and further used:

*Organ Cd (mg kg^-1^ FM) = Organ BTF (d kg^-1^) × Cd daily ingestion (mg d^-1^) [9]*

With BTF equals to 7.79 × 10^-2^ or 8.00 × 10^-1^ kg d^-1^ for kidney, and 8.02 × 10^-2^ or 7.06 × 10^-1^ kg d^-1^ for liver, for 100 or 1000 days of Cd exposure, respectively.

**References**

Beresford NA, Mayes RW, Crout NMJ, Maceachern PJ, Dodd BA, Barnett CL, Lamb CS (1999) Transfer of cadmium and mercury to sheep tissues. Env Sci Tech 33:2395-2402. https://doi.org/10.1021/es9811041

Franz E, Römkens P, Van Raamsdonk L, Van Der Fels-Klerx I (2008) A chain modeling approach to estimate the impact of soil cadmium pollution on human dietary exposure. J. Food Prot 71:2504-2513. https://doi.org/10.4315/0362-028X-71.12.2504

Lin Z, Li M, Wang YS, Tell LA, Baynes RE, Davis JL, Vickroy TW, Riviere JE (2020) Physiological parameter values for physiologically based pharmacokinetic models in food-producing animals. Part I: Cattle and swine. J Vet Pharma Ther 43:385-420. https://doi.org/10.1111/jvp.12861

Phillips CJC, Tudoreanu L (2011) A model of cadmium accumulation in the liver and kidney of sheep derived from soil and dietary characteristics. J Sci Food Agric 91:370-376. https://doi.org/10.1002/jsfa.4195

Prankel SH, Nixon RM, Phillips CJC (2004) Meta-analysis of feeding trials investigating cadmium accumulation in the livers and kidneys of sheep. Environ Res 94:171-183. https://doi.org/10.1016/S0013-9351(03)00084-7

Römkens P, van der Fels-Klerx H, van Raamsdonk L, de Jaeger L, Thielen M, Bosshammer K, Leisner-Saaber J, Rath C, Hamer M (2008) Chain models as a tool to quantify the relation between soil, crop quality and human exposure, Stable tot Table-Geoquality-Part I 198-218

| **Table S1** Metallic trace elements concentrations (mg kg^-1^ dry matter) in organs of *R. japonica* at the end of the experiment (day 41) | | | | | | | | | | | | | | | | | | | | |
| --- | --- | --- | --- | --- | --- | --- | --- | --- | --- | --- | --- | --- | --- | --- | --- | --- | --- | --- | --- | --- |
| Organ | | Rhizome | | | | | | |  | | Stem | |  | Leaf | |  | *P*-value | | |  |
| Soil ^a^ | | T0 ^b^ | | CTL | | POL | |  | | CTL | | POL |  | CTL | POL | SEM | Organ | Soil | Organ × Soil | |
| Metal ^c^ | |  | |  | |  | |  | |  | |  |  |  |  |  |  |  |  | |
|  | Cadmium | | 0.04 ± 0.003 | | 0.04 c | | 0.58 b | |  | | 0.03 d | 1.08 a |  | 0.02 d | 1.02 a | 0.051 | < 0.001 | 0.21 | < 0.001 | |
|  | Copper | | 5.3 ± 0.73 | | 5.3 c | | 6.8 bc | |  | | 9.3 a | 7.4 b |  | 5.7 c | 5.1 c | 0.22 | 0.18 | < 0.001 | < 0.01 | |
|  | Nickel | | 0.8 ± 0.04 | | 1.1 a | | 19.2 b | |  | | 0.7 d | 12.4 b |  | 0.7 d | 26.9 a | 1.03 | < 0.001 | < 0.001 | < 0.001 | |
|  | Zinc | | 8.2 ± 0.20 | | 8.8 d | | 62.4 c | |  | | 11.3 d | 80.7 b |  | 19.9 d | 131.5 a | 3.09 | < 0.001 | < 0.001 | < 0.001 | |
|  | Sulfur | | 815 ± 29.9 | | 769 d | | 868 cd | |  | | 826 cd | 982 bc |  | 1 064 b | 1 407 a | 35.7 | 0.08 | < 0.001 | 0.23 | |
|  | Aluminium | | 343 ± 54.3 | | 210 a | | 295 a | |  | | 30 cd | 19 d |  | 32 bc | 39 b | 13.7 | 0.02 | < 0.001 | 0.34 | |
|  | Iron | | 420 ± 51.8 | | 479 a | | 463 a | |  | | 36 b | 38 b |  | 35 b | 46 b | 19.8 | < 0.001 | < 0.001 | 0.06 | |
|  | Manganese | | 11 ± 0.5 | | 37 b | | 32 b | |  | | 14 c | 25 bc |  | 64 a | 96 a | 7.9 | 0.72 | < 0.001 | 0.03 | |
|  | Calcium | | 10 515 ± 899 | | 8 292 | | 8 173 | |  | | 6 037 | 7 518 |  | 7 952 | 8 490 | 344.4 | 0.02 | 0.16 | 0.37 | |
|  | Phosphorus | | 1 064 ± 17.8 | | 935 c | | 842 c | |  | | 2 812 a | 2 073 b |  | 1 908 b | 1 788 b | 66.3 | < 0.001 | < 0.001 | < 0.01 | |
|  | Potassium | | 4 452 ± 77.1 | | 3 823 b | | 3 290 b | |  | | 9 609 a | 10 736 a |  | 9 934 a | 10 932 a | 416.9 | < 0.001 | 0.19 | 0.41 | |
|  | Magnesium | | 722 ± 26.9 | | 476 c | | 503 c | |  | | 612 c | 873 b |  | 2 087 a | 1 960 a | 36.6 | < 0.001 | 0.25 | 0.02 | |
| a-d: means within a row with different letters differs at *P* ≤ 0.05. | | | | | | | | | | | | | | | | | | | | |
| ^a^ *R. japonica* grow up on soils control (CTL) or polluted with metallic trace elements (POL) | | | | | | | | | | | | | | | | | | | | |
| ^b^ Concentrations (mean ± standard deviation, *n* = 2) in representative sub-samplings of *R. japonica* rhizome at the beginning of the experiment (Time 0, T0). Those data are not included in statistical analyses | | | | | | | | | | | | | | | | | | | | |
| ^c^ Concentrations in As, Pb, Cr and Co were lower than the limit of quantification for 82, 83, 85 and 86 analyses on a total of 87 analyses, respectively | | | | | | | | | | | | | | | | | | | | |
